# Can machine learning-based analysis of multiparameter MRI and clinical parameters improve the performance of clinically significant prostate cancer diagnosis?

**DOI:** 10.1007/s11548-021-02507-w

**Published:** 2021-10-22

**Authors:** Tao Peng, JianMing Xiao, Lin Li, BingJie Pu, XiangKe Niu, XiaoHui Zeng, ZongYong Wang, ChaoBang Gao, Ci Li, Lin Chen, Jin Yang

**Affiliations:** 1 Department of Radiology, Affiliated Hospital of Chengdu University, 82 2nd N Section of Second Ring Rd, Chengdu, 610081 Sichuan Province China; 2 College of Information Science and Technology, Chengdu University, 1 Shiling shang Street, Chengdu, 610106 Sichuan Province China; 3 Department of Pathology, Affiliated Hospital of Chengdu University, 82 2nd N Section of Second Ring Rd, Chengdu, 610081 Sichuan Province China; 4 Department of Urology Surgery, Affiliated Hospital of Chengdu University, 82 2nd N Section of Second Ring Rd, Chengdu, 610081 Sichuan Province China

**Keywords:** Prostate cancer, Radiomics, Texture analysis, Machine learning, Classification, Magnetic resonance imaging

## Abstract

**Purpose:**

To establish machine learning(ML) models for the diagnosis of clinically significant prostate cancer (csPC) using multiparameter magnetic resonance imaging (mpMRI), texture analysis (TA), dynamic contrast-enhanced magnetic resonance imaging (DCE-MRI) quantitative analysis and clinical parameters and to evaluate the stability of these models in internal and temporal validation.

**Methods:**

The dataset of 194 men was split into training (*n* = 135) and internal validation (*n* = 59) cohorts, and a temporal dataset (*n* = 58) was used for evaluation. The lesions with Gleason score ≥ 7 were defined as csPC. Logistic regression (LR), stepwise regression (SR), classical decision tree (cDT), conditional inference tree (CIT), random forest (RF) and support vector machine (SVM) models were established by combining mpMRI-TA, DCE-MRI and clinical parameters and validated by internal and temporal validation using the receiver operating characteristic (ROC) curve and Delong’s method.

**Results:**

Eight variables were determined as important predictors for csPC, with the first three related to texture features derived from the apparent diffusion coefficient (ADC) mapping. RF, LR and SR models yielded larger and more stable area under the ROC curve values (AUCs) than other models. In the temporal validation, the sensitivity was lower than that of the internal validation (*p* < 0.05). There were no significant differences in specificity, accuracy, positive predictive value (PPV), negative predictive value (NPV) and AUC (*p* > 0.05).

**Conclusions:**

Each machine learning model in this study has good classification ability for csPC. Compared with internal validation, the sensitivity of each machine learning model in temporal validation was reduced, but the specificity, accuracy, PPV, NPV and AUCs remained stable at a good level. The RF, LR and SR models have better classification performance in the imaging-based diagnosis of csPC, and ADC texture-related parameters are of the highest importance.

**Supplementary Information:**

The online version contains supplementary material available at 10.1007/s11548-021-02507-w.

## Introduction

Prostate cancer is the second most common type of cancer worldwide. It represents the fifth leading cause of cancer-related death in men globally and the most frequently diagnosed cancer among men in over one-half (105 of 185) of the countries of the world [1]. Over the past decades, despite the relatively low morbidity of prostate cancer in Asian countries, the morbidity and mortality showed a trend of rapid growth as we witnessed a rapid development of the economic and lifestyle changes [2]. In Chinese men, the morbidity of clinically significant prostate cancer (csPC), which is defined as a Gleason score (GS) of 7 or greater [3, 4], is among the highest in Asia [5]. The 10-year survival rate for low-grade prostate cancer is significantly higher than that for csPC [6]. The AUA/ASTRO/SUO guideline recommends active surveillance as the preferable care option for most low-risk localized prostate cancer patients [7]. Therefore, the evaluation of tumor invasiveness has become an important purpose of diagnosis.

As one of the gold standard diagnostic tests, invasive prostate biopsy may lead to discomfort, missed diagnosis, infection and other prostate issues. In recent years, the influence of MRI on prostate cancer diagnosis has rapidly grown, and multiparametric MRI (mpMRI) of the prostate has evolved to be an integral component of the diagnosis, risk stratification and staging process of prostate cancer [8]. Endorsed by the American College of Radiology, the Prostate Imaging Reporting and Data System (PI-RADS version 2.1) [9] stratifies prostate lesions into different categories that reflect their relative likelihood of a csPC [10]. However, some aspects of the criteria for individual sequence scores are still ambiguous. Even for expert readers, it is not uncommon to encounter discrepancies when classifying such terms of PI-RADS [11], which may affect the classification of prostate lesions. Despite the increasing use of mpMRI for prostate cancer diagnosis, radiologists can still miss about 15–30% of all csPCs [12, 13]. Moreover, there is a large inter-observer variability in the interpretation of mpMRI among radiologists [14].

Texture analysis is one of the important methods in radiomics. After high-throughput extraction of massive information from medical images, a large amount of image features are mined on a deeper level. This can provide a more comprehensive and objective characteristic information than naked eye analysis. With the establishment of machine learning (ML) techniques and other predictive models, the state-of-the-art methods showed great potential in prostate cancer detection, tumor stratification and prognosis assessment based on mpMRI. Some studies have used one or more ML models to identify and distinguish benign tumors and malignant prostate cancer, including csPC and non-clinically significant prostate cancer (ncsPC) [15–19]. Although these models may improve the patient quality of life and outcomes, the actual clinical impact and quality of these predictive models may lag behind their expected potential. One reason is that while many models have been developed, only a small number have been more effectively validated, including external validation and temporal validation [20]. As the prediction formula is tailored to the developmental data, and predictive models may correspond too closely or accidentally be fitted to idiosyncrasies in the developmental dataset, known as overfitting, models can perform well on the developmental population but poorly on the external cohort or temporal cohort [21]. In this study, we established ML models combining clinical parameters, texture analysis and dynamic enhanced scanning quantitative parameters. As this study focused on whether differences in the patient cohort factors themselves would affect the stability of the models, in addition to internal validation, temporary validation was used to evaluate their performance in the classification of csPC.

## Materials and methods

### Ethical approval

All procedures performed in studies involving human participants were in accordance with the ethical standards of the institutional research committee and with the 1964 Helsinki declaration and its later amendments or comparable ethical standards. The study has been approved by the Institutional Review Board (IRB) of our institute, and patient consent form was waived because this is a retrospective study with anonymized data.

### Patients

**Fig. 1:**
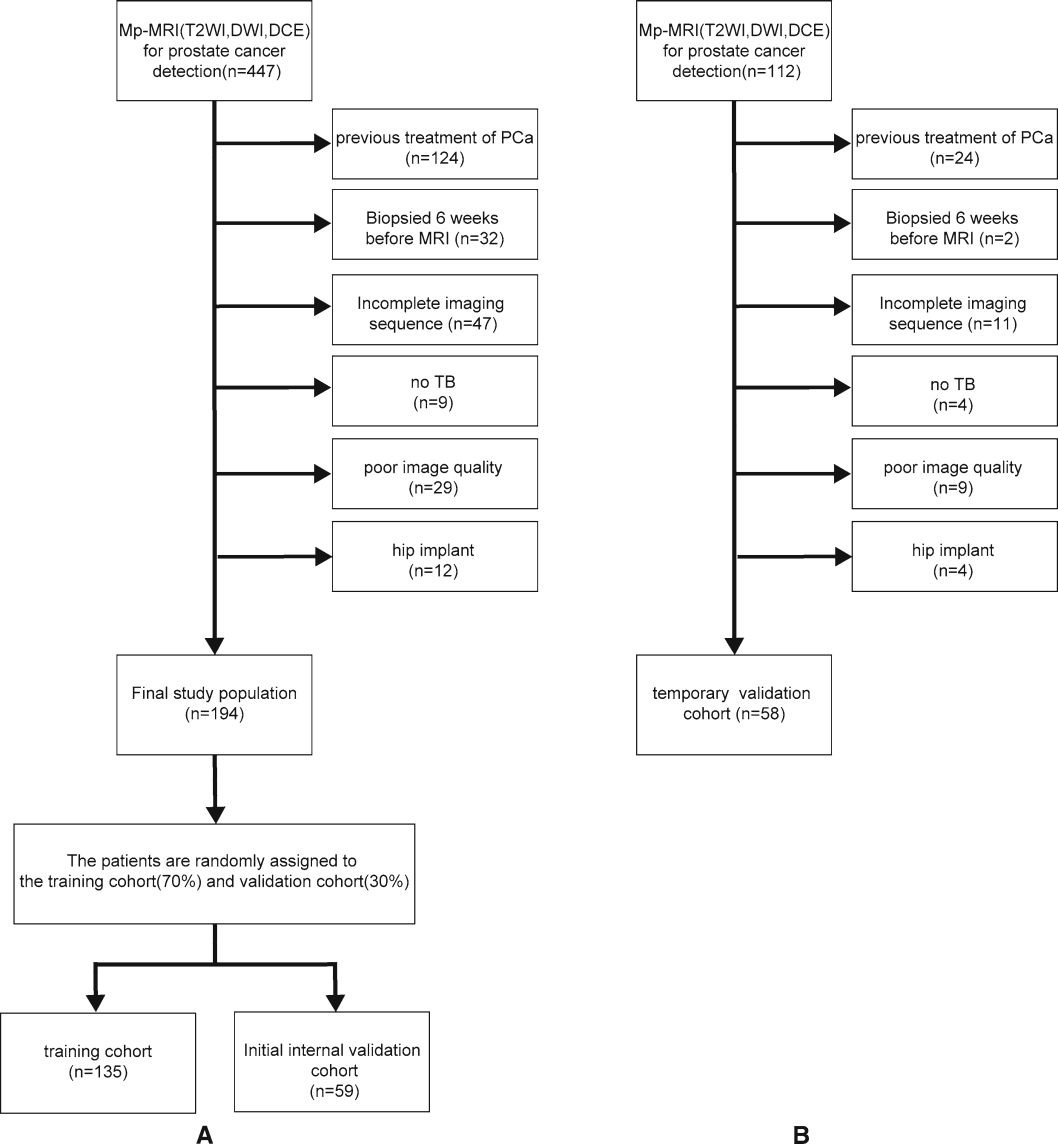
Patient selection details in training cohort and internal validation cohort (A), temporal validation cohort (B). The horizontal arrow represents cases that meet the exclusion criteria to be removed from the study cohort

In this study, we performed retrospective modeling, internal validation and temporary validation. In the first part of retrospective modeling and internal validation, 447 patients who were enrolled from January 2014 to November 2018, with the following inclusion criteria: biopsy-naive status, clinical suspicion of PCa owing to either an elevated PSA level (> 4 ng/mL) or an abnormal DRE (digital rectal examination), complete mpMRI before biopsy, including target biopsy (TB) guided by mpMRI under the PI-RADS v1, v2 or v2.1 system and systemic biopsy in the same procedure. The exclusion criteria included the patients with any previous treatment of PCa, poor image quality or incomplete imaging sequence, marked artifact on MR images attributable to hip implant, or no TB. The selected patients were randomly divided into the training cohort (group 1, 70%, 135 patients) and the internal validation cohort (group 2, 30%, 59 patients). The patient selection is detailed in Fig. 1A.

The above-mentioned inclusion and exclusion criteria were also used in the temporary validation. A total of 112 patients enrolled between January and November 2019 were selected according to the criteria for the temporary validation. The selected patients were assigned to the temporary validation cohort (group 3). Patients in group 3 did not belong to the same dataset as patients in group 1 and group 2 and were completely different in terms of the enrollment time and patient composition. The patient selection is detailed in Fig. 1B. The patient characteristics are detailed in Table 1.

**Table 1:** Descriptive characteristics of the study population

Patient characteristics	Group 1 (*n* = 135)	Group 2 (*n* = 59)	Group 3 (*n* = 58)	*P*-Value
*Age, years*				
Mean(± SD) Median Range IQR	73.88(± 8.74) 75 50–93 69–80	76.62(± 7.9) 78 62–91 71–82.5	72.33(± 8.64) 73 53–90 66.25–78	group 1 vs 2:0.128 group 1 vs 3:0.273 group 2 vs 3:0.008
*tPSA*				
Median Range IQR	18 0.35–300 8.23–57.5	22.9 4.56–300 11.5–91.3	15.3 0.22–159 6.96–32.92	group 1 vs 2:0.073 group 1 vs 3:0.182 group 2 vs 3:0.007
*fPSA*				
Median Range IQR	2.5 0.04–25 1.17–6.265	2.73 0.3–25 1.65–10.75	1.82 0.01–25 1.01–4.99	group 1 vs 2:0.094 group 1 vs 3:0.184 group 2 vs 3:0.006
*V*				
Median Range IQR	53.33 12.13–155.19 33.11–77.17	60.01 13.47–374.4 47.04–89.51	52.80 21.91–198.02 38.70–77.11	group 1 vs 2:0.038 group 1 vs 3:0.000 group 2 vs 3:0.000
*PSAD*				
Median Range IQR	0.351 0.009–12.01 0.136–1.18	0.44 0.04–12.66 0.18–1.46	0.28 0.005–2.83 0.15–0.66	group 1 vs 2:0.343 group 1 vs 3:0.000 group 2 vs 3:0.000
csPC	53	22	18	
ncsPC	82	37	40	
*GS*				
Grade group I (GS0) (GS4) (GS5) (GS6)	82(60.74%) 76(56.3%) 1(0.74%) 0(0%) 5(3.70%)	37(62.71%) 34(57.63%) 0(0%) 1(1.69%) 2(3.39%)	40(68.97%) 39(67.24%) 0(0%) 0(0%) 1(1.72%)	Percentage comparison: group 1 vs 2:0.998 group 1 vs 3:1 group 2 vs 3:1
Grade group II (GS 3 + 4 = 7)	11(8.15%)	7(11.86%)	4(6.90%)	
Grade group III (GS 4 + 3 = 7)	8(5.93%)	0(0%)	4(6.90%)	
Grade group IV (GS8)	6(4.44%)	5(8.47%)	6(10.34%)	
Grade group V (GS9) (GS10)	28(20.74%) 20(14.81%) 8(5.93%)	10(16.95%) 9(15.25%) 1(1.69%)	4(6.90%) 3(5.17%) 1(1.72%)	

group 1, Training cohort; group 2, internal validation cohort; group 3, temporary validation cohort; IQR, interquartile range; SD, standard deviation; tPSA, total prostate-specific antigen; fPSA, free prostate-specific antigen; V, volume of prostate measured on MRI; PSAD, prostate-specific antigen density; csPC, number of csPC; and ncsPC, number of ncsPC

### Multiparametric MRI

All imaging was performed using a 1.5 T system (Magnetom Avanto, Siemens Healthcare) with a combined spine-array coil and a body-array receive-only coil (Tim Trio, Siemens Healthcare). None of the patients underwent bowel preparation or received butylscopolamine bromide. The scan sequences included high-resolution axial T2-weighted imaging, diffusion-weighted imaging (DWI) and DCE-MRI. The MRI parameters are listed in Supplementary Table S1.

### Biopsy procedure and histopathologic examination

The standard 12-core systematic biopsy and TB were performed under the guidance of transrectal ultrasound (TRUS). Each core specimen was placed in a specific location of the prostate biopsy collection kits according to the prostate region that it came from. These biopsies included at least two additional cores for each target, and TB was recognized by cognitive registration based on the zonal anatomy or imaging landmarks, such that a urologist was needed to accurately associate real-time ultrasound images with the target lesions in the MRI images, and an experienced uroradiologist (6 years of experience in prostate MRI) helped the urologist to identify the details in and around the target lesion (prostate shape, verumontanum position, distance from the apex or the prostate base, presence of benign cyst or calcification nearby). The lesions with a GS ≥ 7 were defined as csPC.

### ROI for image texture analysis and DCE-MRI quantitative analysis

**Fig. 2:**
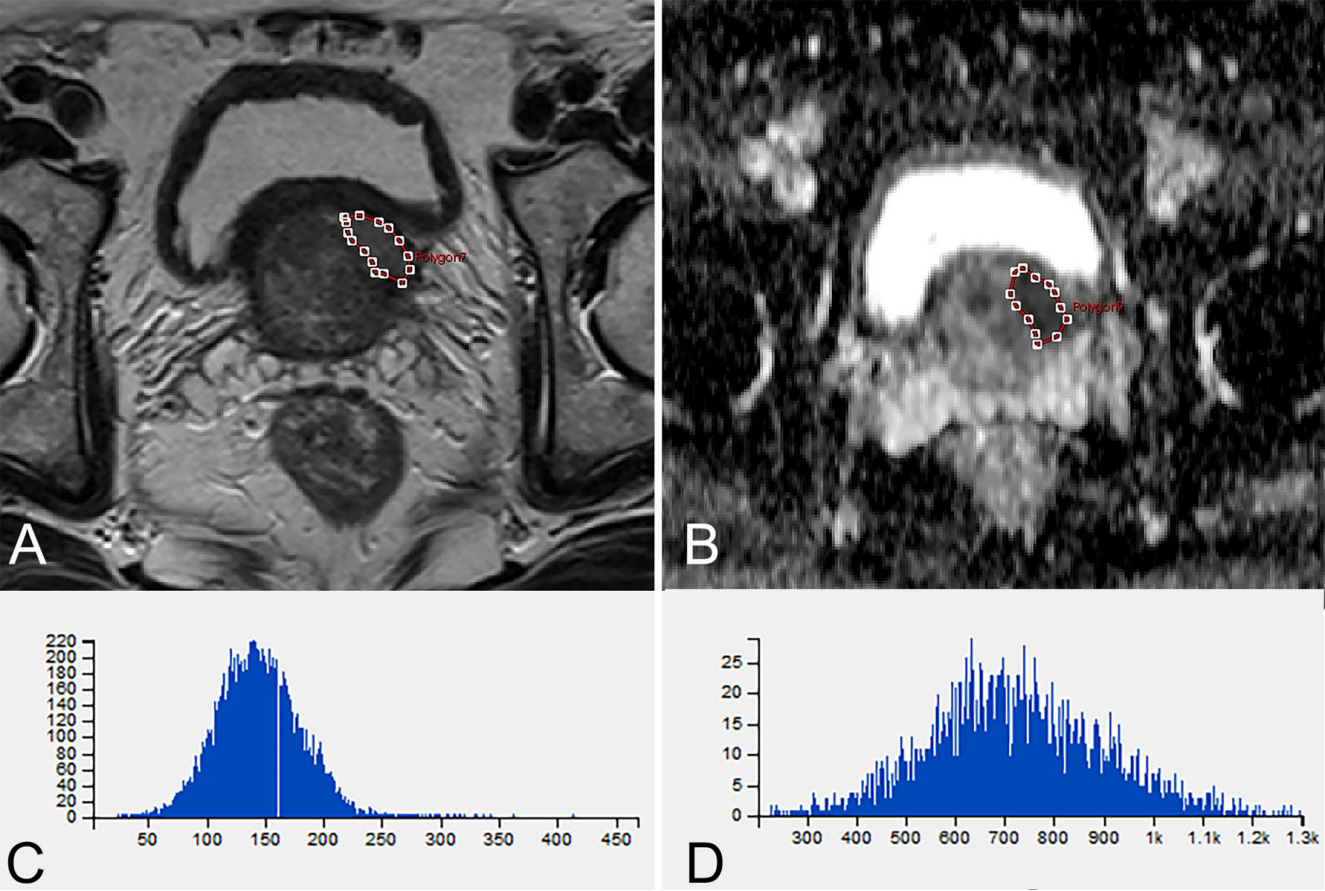
A 66-year-old man with Gleason score 4 + 3 = 7 lesion. **A** T2-weighted MR image shows ROI of lesion (red) manually drawn for transition zone. **B** ADC mapping image shows ROI of lesion (red) manually drawn for transition zone. **C** Histogram of A. **D** Histogram of B

In the first part of modeling and internal validation, texture analysis (TA) was performed by one radiologist with 10 years of experience in prostate MRI, who assessed on the T2-weighted, diffusion-weighted and DCE MR images and independently performed TA using the Omni-Kinetics software (version 2.01, GE Healthcare). The regions of interest (ROIs) covering the entire tumor area were manually delineated on each axial slice based on the pathological results (Fig. 2). The ROIs of lesion layers were merged into a 3D ROI. The ROIs should be delineated on the main lesions, which were defined as the ones with the highest GS or most aggressive features [4]. Necrotic areas, cystic degeneration, hemorrhage, calcification, as well as the urethra, bladder, seminal vesicle and vascular nerve bundles should be as far as possible avoided. If a lesion turns out to be csPC, then it would be selected as a ROI. The areas with ncsPC, low-grade carcinoma, high-grade prostatic intraepithelial neoplasia or similarity to the manifestation of prostate cancer were defined as ROIs. For DCE-MRI, the extended Toft’s model (ETM) was implemented for the quantitative analysis of microcirculation.

In the part of temporal validation, the ROIs were independently delineated by two radiologists (Doctor A: 10 years and Doctor B: 3 years of experience in prostate MRI) who were blind to the experiment following the above-described method, but without the pathological results to accord with when delineating.

### Machine learning modeling and statistical analysis

The SPSSAU.com online tool was used for the statistical analyses of descriptive characteristics of the study population and the intra-class correlation (ICC) analysis of temporal validation. The other parts of the study, including modeling, validation, etc., were performed using the R language (version 3.63) programs. Feature extraction was also performed using the O.K. software. The types of the computer-derived features included first-order parameters, gradient-based histogram features, gray-level co-occurrence matrix (GLCM), run-length matrix (RLM), DCE-MRI quantitative features (extendedtofts_linear algorithm) and clinical parameters including the age, tPSA, fPSA, prostate volume and PSAD, which were calculated based on the voxels in the delineated ROIs (Supplementary Table S2). Although 233 features were extracted, not all of them were helpful in predicting csPC. Therefore, the R language programs were used to reduce the dimensionality, standardize the data and select the features. Logistic regression (LR), stepwise regression (SR), classical decision tree (cDT), conditional inference tree (CIT), random forest (RF) and support vector machine (SVM) models were constructed, and their diagnostic efficacy was compared with the receiver operating characteristic curve (ROC) and confounding matrix. Delong’s method was used to compare the difference in the area under the ROC curve (AUC), and a *P* < 0.05 was considered to be statistically significant. Finally, the models were validated using the data of the temporal validation group (*n* = 58), and the differences in the sensitivity, specificity, negative predictive value, positive predictive value and AUC of different models were compared.

## Results

### Patient characteristics

The ML modeling and internal validation included 194 male patients, while the temporal validation cohort included 58 male patients. The parameters of age, tPSA and fPSA significantly differed between groups 2 and 3. The volume of the prostate and PSAD significantly differed between groups 1 and 3, as well as between groups 2 and 3. There was no significant difference in the composition ratio of GS among the three groups (Table 1).

### Model construction and internal validation evaluation

**Fig. 3:**
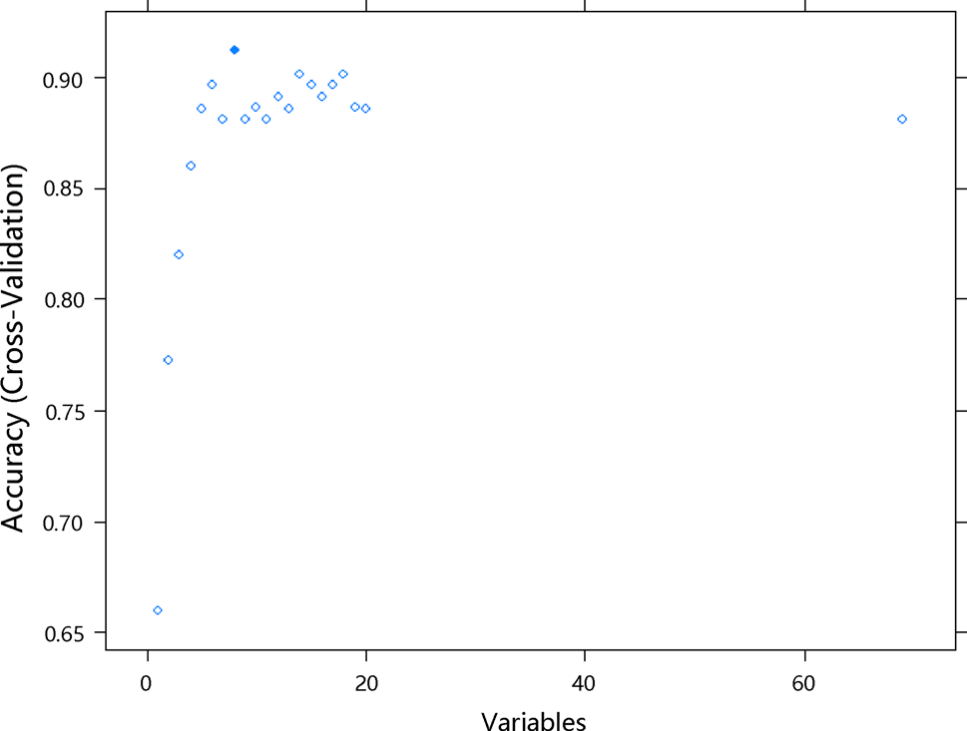
Counting from left to right on the horizontal axis, the solid blue dot is at the 8th position. It means that after dimension reduction and feature selection, eight independent variables were obtained. The X-axis represents the number of variables, and the Y-axis represents accuracy

After dimensionality reduction and feature selection, the following eight independent variables were obtained: ADC.Quantile95, ADC.MinIntensity, ADC.uniformity, PSAD, T2.RelativeDeviation, Vp0.1, T2.Variance and T2.Quantile5 (Fig. 3) (Supplementary Table S3*)*.

**Fig. 4:**
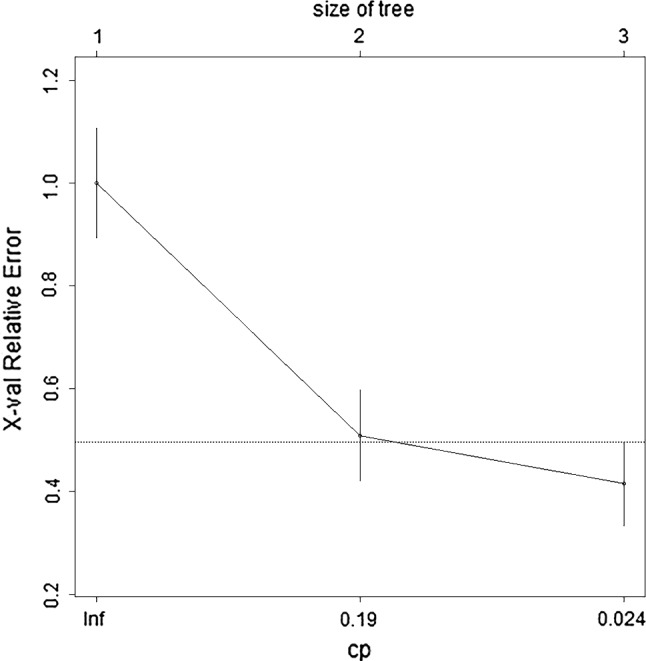
In cDT model building, the cross-validation error and complexity parameter diagram are displayed. The abscissa is the complexity parameter, and the ordinate is the cross-validation error. For all the trees with cross-validation errors within one standard deviation of the minimum cross-validation error, the tree with the lowest CP value is the optimal tree. The tree corresponding to the left-most CP value below the dotted line should be selected. The optimal tree is the tree divided twice (three terminal nodes)

**Fig. 5:**
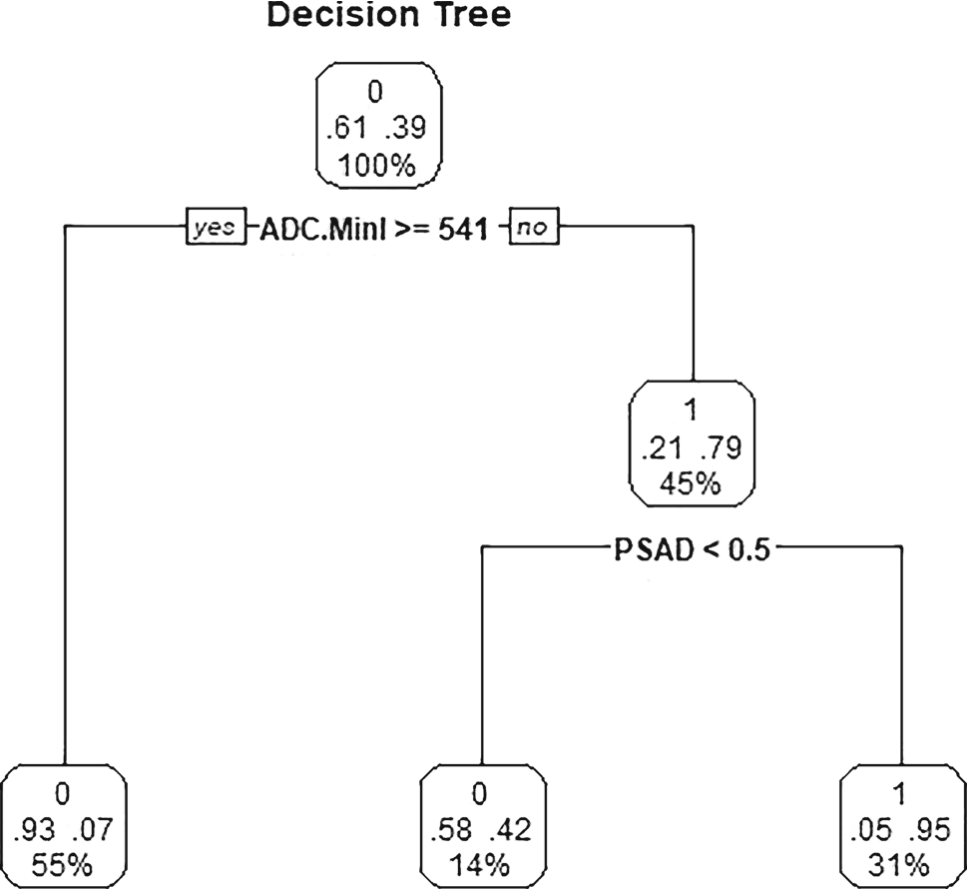
Classical decision tree model. The optimal tree was the tree divided twice (three terminal nodes). Start at the top of the tree, go down to the left if the condition is true, otherwise go down to the right, and the classification ends when the observation point reaches the terminal node. Each node has the probability of the corresponding category and the proportion of the sample

**Fig. 6:**
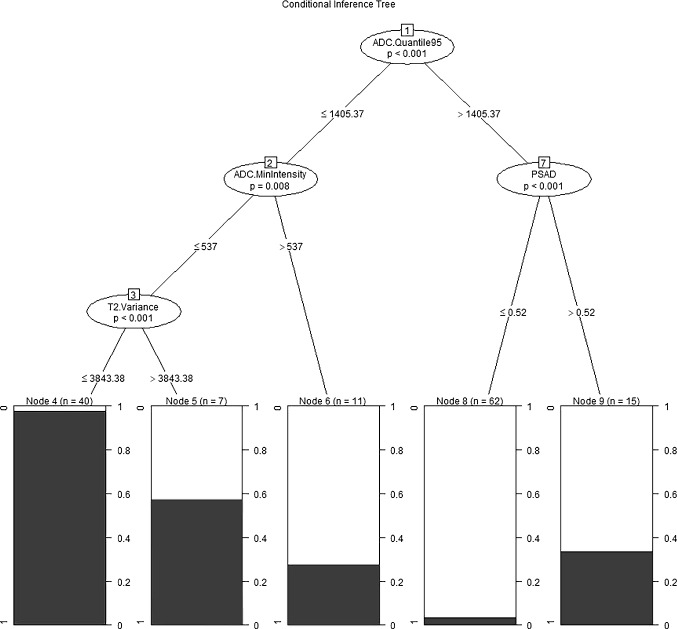
Conditional inference tree model. Start at the top of the tree, classify the two opposite conditions, one on the left and one on the right, and then classify more conditions. The shaded area in each node represents the corresponding csPC proportion, and the figure indicates the number of cases that meet the corresponding criteria

**Fig. 7:**
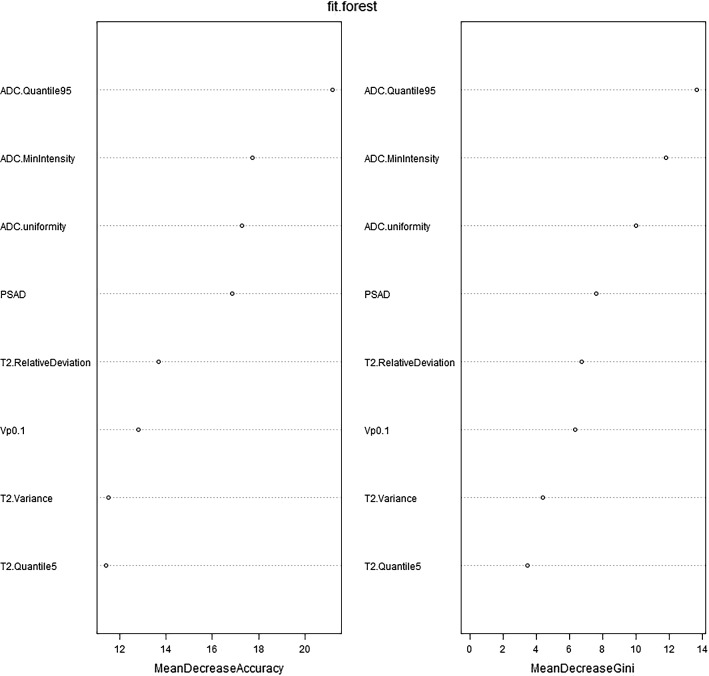
The importance ordering of independent variables of RF model is shown. MeanDecreaseAccuracy and MeanDecreaseGini indicate the importance of the variable. MeanDecreaseAccuracy: The degree to which the prediction accuracy of random forest decreases when the value of a variable is changed to a random number. The larger the value, the more important the variable. MeanDecreaseGini: The influence of each variable on the heterogeneity of observed values at each node of the classification tree was calculated to compare the importance of variables. The larger the value, the more important the variable

**Fig. 8:**
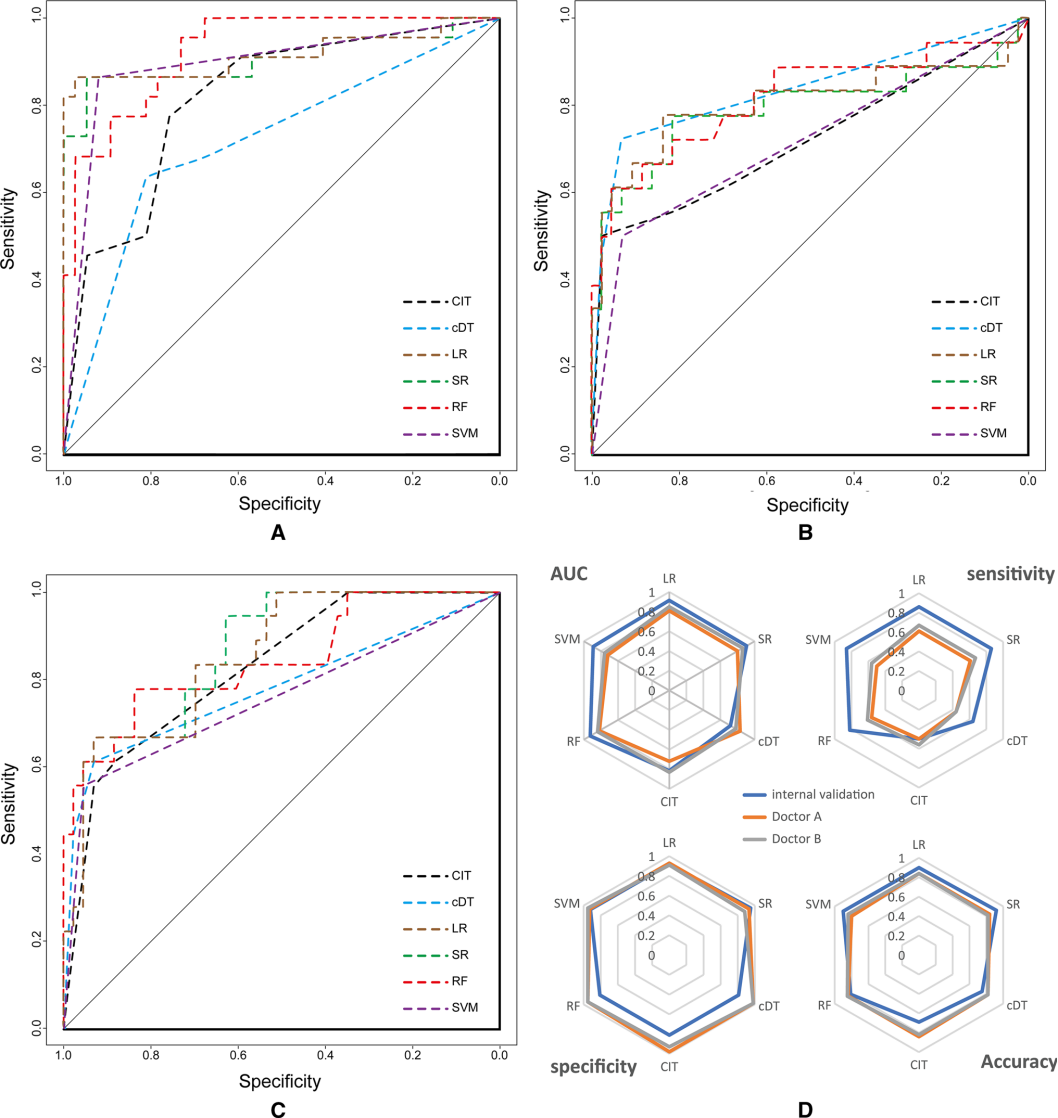
The ROC of LR, SR, cDT, CIT, RF and SVM models in the internal validation cohort are shown in A-C. AUC of ROC, sensitivity, specificity and accuracy for internal and temporal validation (two doctors) are shown on the radar chart in D. A-C: RF, LR and SR models have been relatively stable in the larger AUC during internal validation and temporal validation. In D, the blue line represents internal validation, and the orange and gray lines represent the ability of the machine learning model to classify the ROI delineated by the two radiologists. The orange line and gray line can be observed to almost coincide. The sensitivity of the machine learning model was reduced, but the AUC, specificity and accuracy were stable at a good level

The independent predictors screened by the LR model were t2.Variance, ADC.Quantile95, Vp0.1 and ADC.MinIntensity (*P* < 0.05). The independent predictors of the SR model were T2.RelativeDeviation, ADC.MinIntensity, T2.Variance, ADC.Quantile95 and Vp0.1 (*P* < 0.05). In addition, the *P* values of ADC.Quantile95 and ADC.MinIntensity were less than 0.01. In the cDT model building, the cross-validation error and complexity parameter diagram are displayed in Fig. 4. The optimal tree was the tree divided twice (three terminal nodes) (Fig. 5). The CIT model is shown in Fig. 6, and the ordering of independent variables of the RF model according to importance is shown in Fig. 7. Figure 8 shows the ROC of the LR, SR, cDT, CIT, RF and SVM models in the internal validation cohort. Table 2 shows the diagnostic predictive features of the models of the internal validation cohort. The models with statistically significant differences in the AUC are displayed in Table 4. There were 7 cases with a GS = 7 in the internal validation cohort. Three cases were correctly classified by all ML models. Two cases were correctly classified by some of the ML models (DT, CIT misjudged twice; RF misjudged once). Two cases were misclassified by all ML models (Table S4).

**Table 2:** Diagnostic predictive features of the models of the internal validation cohort

	Sensitivity	Specificity	PPV	NPV	Accuracy	AUC	YI
LR	0.86	0.92	0.86	0.92	0.9	0.915	0.78
SR	0.86	0.95	0.90	0.92	0.92	0.905	0.81
cDT	0.64	0.81	0.67	0.79	0.75	0.717	0.45
CIT	0.50	0.81	0.61	0.73	0.69	0.815	0.31
RF	0.82	0.81	0.72	0.88	0.81	0.925	0.63
SVM	0.86	0.92	0.86	0.92	0.9	0.891	0.78

PPV: positive predictive value; NPV: negative predictive value; and YI: Youden index

### Temporal validation evaluation

**Fig. 9:**
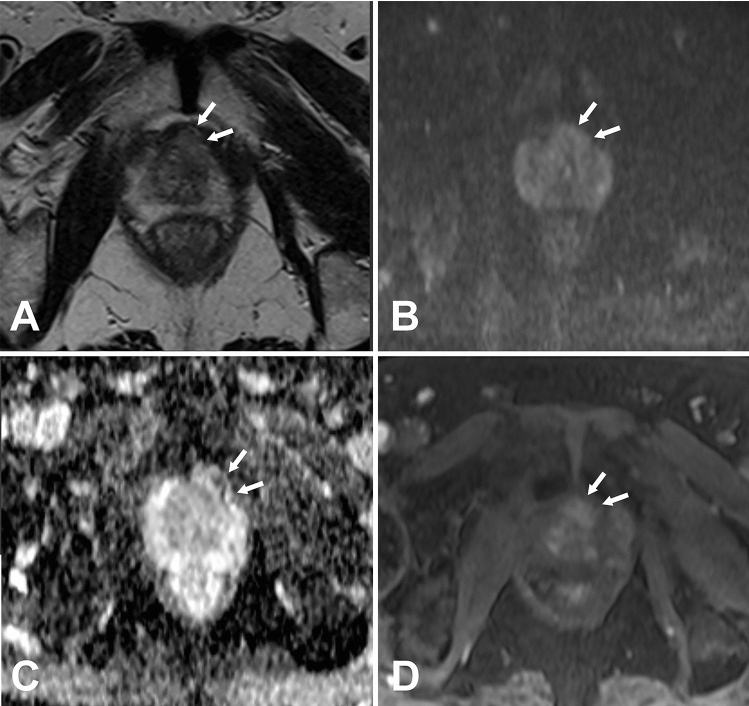
A 78-year-old man, the machine learning models misjudged 3 + 4 = 7 lesion (PI-RADS 4) in the transitional zone at the apex of the prostate (white arrow). The lesion showed slightly low signal on T2WI, no significantly high sign on DWI (*b* = 800), uneven signal reduction on ADC and positive dynamic contrast enhancement with early enhancement

**Fig. 10:**
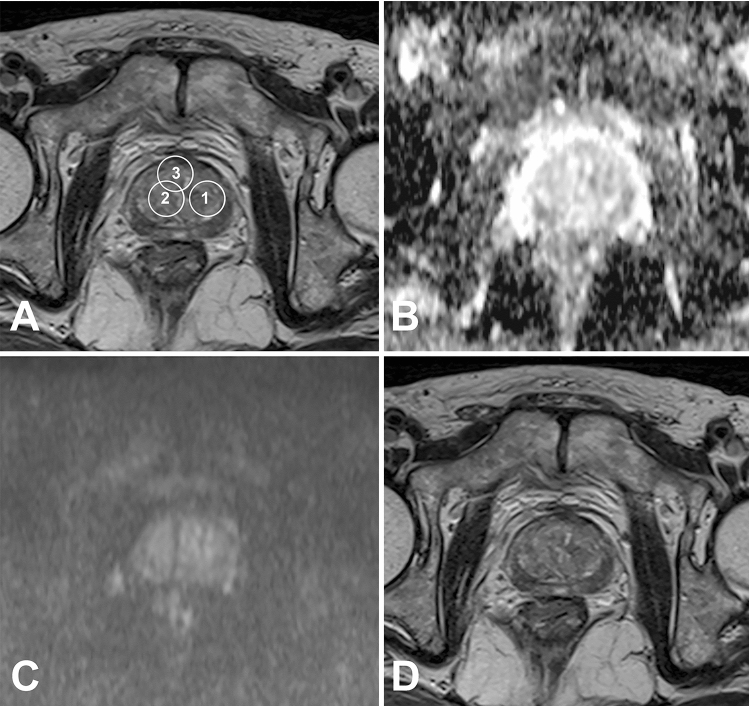
A 74-year-old man; only one core in the left TZa zone (No. 1 in A) had a GS of 4 + 3 = 7. The lesion accounted for 2% of the tissue strips, among which 4 score components accounted for 60% (1.8% of the tissue strips) and 3 score components accounted for 40% (0.8% of the tissue strips). The GS of the two cores from the right TZa and AFS (No. 3 and 2 in A, respectively) is 3 + 3 = 6, accounting for 4% and 1% of the tissue strips, respectively. No lesion with a PI-RADS score ≥ 3 could be found in the MR images, and the ML models misjudged

In the temporal validation, intra-class correlation (ICC) analysis was used to measure the degree of agreement across the raters on each of the 8 important variables. High concordance was found between the two observers in T2.RelativeDeviation, ADC.MinIntensity, T2.Quantile5 and ADC.uniformity, particularity in the last two, while a poor internal consistency was found in ADC.Quantile95, Vp0.1 and T2.Variance. The sensitivity of the temporal validation was lower than that of the internal validation (*P* < 0.05). The specificity, NPV, PPV, accuracy, AUC and YI of temporal validation were not significantly different from those of internal validation (*P* > 0.05). The classification ability of these models for GS > 7 cases was better than the cases with a GS = 7, and the classification performance remained stable for both GS > 7 and GS = 7 cases. There were 8 cases with a GS = 7 in the temporal validation cohort. Three cases were correctly classified by all ML models. Compared with the pathological results, the lesions with a PI-RADS score of 4–5 of these 3 cases could be found in the MR images (Fig. 2). The 5 cases that were misjudged by different models were the same (Table S4). In one case, the machine learning models misjudged 3 + 4 = 7 lesion (PI-RADS 4) in the transitional zone at the apex of the prostate. The lesion showed slightly low signal on T2WI, no significantly high signal on DWI (*b* = 800), uneven signal reduction on ADC and positive dynamic contrast enhancement with early enhancement (Fig. 9). No lesion of these 4 remaining cases with a PI-RADS score ≥ 3 could be found in the MR images (Fig. 10). When the cases with a GS of 7 were removed from the temporal validation database, the sensitivity and Youden index of all the models increased to varying degrees, as shown in Table 3. There was no change in the specificity of the models before and after removing the cases with a GS of 7, the PPV was slightly reduced, and the NPV was marginally increased.

**Table 3:** Comparison of the predictive features of the temporary validation

	Sensitivity/sensitivity *	Specificity/specificity *	PPV/PPV*	NPV/NPV*	Accuracy/Accuracy *	AUC/AUC*	YI/YI *
*Doctor A*							
LR	0.61/0.89	0.93/0.93	0.79/0.73	0.85/0.98	0.84/0.92	0.81/0.97	0.54/0.82
SR	0.61/0.89	0.93/0.93	0.79/0.73	0.85/0.98	0.84/0.92	0.80/0.97	0.54/0.82
cDT	0.44/0.56	0.98/0.98	0.89/0.83	0.81/0.91	0.82/0.90	0.83/0.92	0.42/0.54
CIT	0.50/0.67	0.98/0.98	0.90/0.86	0.82/0.93	0.84/0.92	0.72/0.82	0.48/0.65
RF	0.56/0.78	0.95/0.95	0.83/0.78	0.84/0.95	0.84/0.92	0.82/0.96	0.51/0.73
SVM	0.50/0.67	0.93/0.93	0.75/0.67	0.82/0.93	0.8/0.88	0.72/0.80	0.43/0.6
*Doctor B*							
LR	0.67/1	0.91/0.91	0.75/0.69	0.87/1	0.84/0.92	0.85/0.97	0.58/0.91
SR	0.67/1	0.88/0.88	0.71/0.64	0.86/1	0.82/0.9	0.86/0.97	0.55/0.88
cDT	0.44/0.56	0.98/0.98	0.89/0.83	0.81/0.91	0.82/0.9	0.78/0.92	0.42/0.54
CIT	0.56/0.78	0.93/0.93	0.77/0.7	0.83/0.95	0.82/0.9	0.83/0.92	0.49/0.71
RF	0.61/0.89	0.95/0.95	0.85/0.8	0.85/0.98	0.85/0.94	0.84/0.97	0.56/0.84
SVM	0.56/0.78	0.95/0.95	0.83/0.78	0.84/0.95	0.84/0.82	0.76/0.87	0.51/0.73

^*^Gleason score 7 cases were removed

## Discussion

In this study, mpMRI-TA, DCE-MRI quantitative analysis and clinical parameters were combined in a compound database. Eight important variables with the highest prediction accuracy were obtained after the feature selection and dimensionality reduction, and then, ML modeling and the evaluation of the models were carried out. According to Tables 3 and 4, the RF, LR and SR models have higher diagnostic abilities for csPC, and the overall performance of the SVM model in the temporal validation slightly declined. The performance of the RF, LR and SR models was relatively stable, not only in the unblinded internal validation, but also the blinded temporal validation, even though the number of experience years of the two doctors in the temporal validation greatly differed. Due to the existence of overfitting, prediction models may correspond too closely or accidentally be fitted to idiosyncrasies in the development dataset. In the three groups in this study, the differences in the baselines of some clinical parameters were statistically significant, but the classification ability of the machine learning models for different validation sets was still relatively stable, indicating that these models still had good universality when dealing with different datasets.

**Table 4:** Models with statistically significant difference in AUC

Validations	Comparison of models	*P*
1. Internal validation		
	RF VS. cDT	0.000
	CIT VS. cDT	0.04
	SVM VS. cDT	0.001
	SR VS. cDT	0.002
	LR VS. cDT	0.001
	RF VS. CIT	0.005
2. temporary validation		
2.1 Doctor A		
	cDT VS. SVM	0.027
2.2 Doctor A(GS 7 cases removed)		
	RF VS. SVM	0.016
	SR VS. SVM	0.018
	LR VS. SVM	0.018
2.3 Doctor B		
	CIT VS. SVM	0.041
	SR VS. SVM	0.018
	LR VS. SVM	0.031
	SR VS. DT	0.046
2.4 Doctor B(GS 7 cases removed)		
	RF VS. CIT	0.048

In the temporal validation, the sensitivity of each model was lower than that of the internal validation. The specificity, accuracy, PPV, NPV and AUC remained at a good level, similar to the internal validation. The analysis of the validations showed that the main misjudgments were in the cases with a GS of 3 + 4 = 7. When all the cases with a GS of 7 were removed from the database and verification was performed again, the sensitivity significantly increased to a level similar to that of internal validation. The reasons were analyzed as follows: (1) inherent differences between temporal and internal validation cohorts. Cases with 7 scores in the internal validation cohort accounted for 31.81% of csPC, while in the temporal validation cohort, cases with a score of 7 accounted for 44.44% of csPC, and the classification of cases with GS = 7 was difficult for ML-based classification. (2) Some lesions were too small. (3) Lesions are not typical on T2WI or DWI, and the corresponding texture data may not be adequate to be correctly classified by ML models. Our findings proved that ROI delineation is not the main cause of ML models misjudgment, since we repeated the experiment on 5 cases of misjudgment by ML models in the temporal verification group and the results remained unchanged. Small foci of the disease may be occult on mpMRI due to the limitations of the technology to resolve small nests of prostate cancer < 0.5 cc in volume, or due to a sparsely distributed tumor interspersed between the normal prostatic stroma [22, 23]. The study of Rozenberg et al. showed that the quantitative ADC measurements and individual ADC texture features had a limited performance in predicting GS upgrading of 3 + 4 = 7 cancers and identifying medium-risk tumors. Logistic regression models with several texture features can improve the prediction accuracy [24]. Further studies are needed to evaluate the ability of ADC texture analysis to identify moderate-risk tumors. Considering the active surveillance, the proportion of GS 3 + 4 = 7 tumors may be one of the important factors. Since the long-term prognosis of GS 3 + 4 = 7 tumors is significantly different from that of GS 4 + 3 = 7 prostate cancer, it is increasingly important to distinguish between the two types of tumors with different GS [25].

The RF model is a classifier that contains multiple decision trees. RF has shown an excellent classification performance for processing balanced sample sets, with few adjustment parameters and good noise tolerance. Besides, it is not prone to overfitting and can efficiently process a large number of features [26]. Nathan Lay et al. established an RF model and an SVM model based on MRI signals and texture features. Their results showed that the RF model with an AUC of 0.93 was superior to the SVM model with an AUC of 0.86 [27], which is basically consistent with the corresponding results of our study. SVM has shown a high performance in processing data that are nonlinear or have a small number of samples or high dimensions [28]. SVM classifiers developed in combination with the PI-RADS scores and MR radiomics features have made significant advances in the diagnosis of prostate cancer in several recent studies [29]. In this study, SVM classifiers performed well in the internal validation, but had a slightly lower performance in the temporal validation. LR is one of the most commonly used ML algorithms in dichotomous tasks. It is a supervised learning ML algorithm that is widely used due to its ease of use and interpretability [30]. Some predictive variables based on LR that do not pass the significance test are removed, and then, the SR model is obtained. In our study, since the database was selected for dimensionality reduction and feature selection in advance, only a few variables with a high prediction accuracy were retained. The results of our study showed that there was no significant difference in the diagnostic efficiency between the LR and SR models. The cDT model is a common model for data mining, which is based on binary output variables and a set of predictive variables. CIT is a variant of cDT, but the variable selection and ROI delineations are based on significance tests rather than intra-group homogeneity. The cDT was built using the rpart software package, and CIT was built using the Party package and does not require pruning. The threshold determines the complexity of the model. Decision tree models are prone to overfitting [31], but RF models basically avoid this problem.

In this study, the RF model ranked the parameters according to their importance, and the top five items in the charts of MeanDecreaseAccuracy and MeanDecreaseGini were ADC.Quantile95, ADC.MinIntensity, ADC.uniformity, PSAD and T2.RelativeDeviation. The first three parameters are all related to the ADC texture analysis. PSAD is a derived parameter of PSA that represents the PSA content of the prostate per unit volume. It has already been shown that the diagnostic accuracy can be increased by adding the PSA density levels to the diagnostic process [3, 32]. Adding the parameters of age and PSA density to the PI-RADS scores improves the diagnostic accuracy for csPCa. A combination of these variables with PI-RADS v2 can help to avoid unnecessary in-bore biopsies while still detecting the majority of csPC lesions [33]. The T2WI relative texture parameter T2.RelativeDeviation was ranked 5th in the importance ranking, while Vp0.1, a parameter derived from the DCE-MRI quantitative analysis, was ranked 6th. Vp represents the percentage of the intravascular contrast medium volume, reflecting the characteristics of the increased average vascular density, increased perfusion vessels and high vascular permeability.

This study has some limitations as follows. First, since the sample size was relatively small, the convolutional neural network model was not selected for evaluation. Second, part of the pathological results came from the ultrasound-guided puncture biopsy, which could not avoid the possibility of missed puncture diagnosis. Third, no specific study has been specifically performed for a large number of cases with a GS of 7, which will be covered in the next study. Fourthly, manual segmentation was adopted in this study, and 3D automatic segmentation with higher efficiency based on deep learning of csPC will be performed in the following study [34].

## Conclusions

Machine learning models have a good classification ability for csPC. Compared with internal validation, the sensitivity of each model in temporal validation was reduced, but the specificity, accuracy and area under the ROC curve remained stable at a good level. The RF, LR and SR models have a better classification performance in imaging-based diagnosis of csPC, and ADC texture-related parameters are among the parameters with the highest importance.

## Supplementary Information

Below is the link to the electronic supplementary material.

Supplementary file1 (DOCX 21 kb)

## Data Availability

The data that support the findings of this study are available from the corresponding author on reasonable request.

## Code availability

The custom code of this study is available from the corresponding author on reasonable request.
