# Supplementary material for: Can machine learning-based analysis of multiparameter MRI and clinical parameters improve the performance of clinically significant prostate cancer diagnosis?
Source: Int J Comput Assist Radiol Surg. 2021 Oct 22;16(12):2235–49. doi: 10.1007/s11548-021-02507-w (PMC8616865; doi:10.1007/s11548-021-02507-w)
Supplement: Supplementary file 1 — Supplementary file1 (DOCX 21 kb) [file 11548_2021_2507_MOESM1_ESM.docx]

**Supplementary**

**Table *S1*** Magnetic resonance imaging sequence parameters

|  | Sequence Type | TR/TE | slice thickness  (mm) | interslice gap(mm) | flip angle  (degree) | matrix size | FOV(mm) | NSA |
| --- | --- | --- | --- | --- | --- | --- | --- | --- |
| T2WI | TSE | 4000/101 | 3 | 0.6 | 150 | 256x 205 | 200x200 | 1 |
| DWI  (b=0,100,800) | SE-EPI | 3700/80 | 3.6 | 0 | 90 | 160x 95 | 260x 221 | 6 |
| DCE-MRI | VIBE | 5/1.69 | 3.6 | 0 | 12 | 192x138 | 260x260 | 1 |

TR, echo time; TE, repetition time; FOV, field of view; T2WI, T2-weighted imaging; DWI, diffusion-weighted imaging; DCE-MRI, dynamic contrast-enhanced MRI;NSA, number of signals averaged.

**Table *S2*** Parameters of various categories for machine learning modeling

| **category** | **parameters** |
| --- | --- |
| Clinical parameters: | age, TPSA, fPSA, MRI volume, PSAD |
| T2WI Histogram Parameters  ADC Histogram Parameters  DCE Histogram Parameters | **First Order Parameters:**  MinIntensity, MaxIntensity, MedianIntensity, MeanValue, stdDeviation, Variance, VolumeCount, VoxelValueSum, RMS, Range, MeanDeviation, RelativeDeviation, MinLocation, MaxLocation  **Histogram Parameters:**  skewness, kurtosis, uniformity, Energy, Entropy, FrequencySize, Quantile5, Quantile10, Quantile25, Quantile50, Quantile75, Quantile90, Quantile95  **GLCM Parameters:**  GlcmBinSize, GlcmTotalFrequency, GlcmMatrixMean, GlcmRelativeFrequency, Energy, Entropy, Inertia, Correlation, InverseDifferenceMoment, ClusterShade, ClusterProminence, HaralickCorrelation, InvalidFeatureName  **RLM Parameters:**  MinIntensity, MaxIntensity, NumberOfIntensityBins, MinSize, MaxSize, NumberOfSizeBins, ShortRunEmphasis, LongRunEmphasis, GreyLevelNonuniformity, unLengthNonuniformity, LowGreyLevelRunEmphasis, HighGreyLevelRunEmphasis, ShortRunLowGreyLevelEmphasis, ShortRunHighGreyLevelEmphasis, LongRunLowGreyLevelEmphasis, LongRunHighGreyLevelEmphasis |
| ExtendedTofts Linear Parameters | Image, Ktrans, Kep, Ve, TTP, MAX Conc, AUC, MAXSlope  (Min, Max, Median, Mean, Std, Area,10%, 25%, 50%, 75%, 90%) |

**Table *S3*** Descriptions of independent variables

| Independent Variables | Descriptions |
| --- | --- |
| ADC.Quantile95 | The value at the 95th percentile in the normal distribution curve formed after transformation in ADC image texture. |
| ADC.MinIntensity | The minimum signal strength of ADC histogram texture feature. |
| ADC.uniformity | The uniformity of ADC histogram texture features. |
| PSAD | tPSA divided by prostate volume (MRI measurement). |
| T2.RelativeDeviation | The relative deviation of T2WI image histogram texture features. |
| Vp0.1 | The value corresponding to the 10th percentile voxel after ranking from small to large volume fraction of plasma (Vp) in ROI. |
| T2.variance | The gray-level co-occurrence matrix (GLCM) texture feature Variance of T2WI images. |
| T2.Quantile5 | The value at the 5th percentile of the normal distribution curve formed after transformation in T2WI texture. |

| **Table *S4*** The judgment and pathological information of cases with a GS of 7 in internal validation and temporal validation of ML models | | |
| --- | --- | --- |
|  | Internal validation | Temporal validation |
| All correct | 3 | 3 |
| Partial correctness | 2 (DT, CIT misjudged twice; RF misjudged once) | 0 |
| All misjudged | 2 | 5 |
| Pathological information of cases correctly classified by all ML models | All the three cases were 3+4=7 (4 cores). The lesions with a PI-RADS score of 4-5 of these 3 cases could be found in the MR images. | One case was 4+3=7 (3 cores), 3+4=7 (1 core), 3+3=6 (1 core), another was 3+4=7 (2 cores), 3+3=6 (4 cores), and a third was 3+4=7 (4 cores), 3+3=6 (4 cores). The lesions with a PI-RADS score of 4-5 of these 3 cases could be found in the MR images (Fig. 2). |
| Pathological information of cases correctly classified by a some ML models | Both cases were 3+4=7, one case that had no lesion with PI-RADS > 3 could be found on MRI, the lesion accounted for 10% of the tissue strips. The lesion of the other case was located at the apex of the prostate, the lesion accounted for 5% of the tissue strips. |  |
| Pathological information of cases that were misclassified by all ML models | Both cases were 3+4=7 (2 core). In one case, csPC was detected in only one core, and the lesion accounted for 80% of the tissue strips. In the other case, the lesion was located at the AFS zone of the prostate. The initial pathological report of the core was atypical small acinar hyperplasia, which was reported as 3+4=7 after immunohistochemistry (IHC). The lesion accounted for 2% of the tissue strips. | There were 5 cases of misjudgment, 4+3=7 (1 core), 3+4=7 (5 cores) and 3+3=6 (8 cores). According to the pathological results, in one case, the machine learning models misjudged 3+4=7 lesion (PI-RADS 4) in the transitional zone at the apex of the prostate (Fig. 9). No lesion of the 4 remaining cases with a PIRADS score ≥ 3 could be found in the MR images (Fig. 10). The initial pathological report of the core of 4+3=7 was high grade prostatic intraepithelial neoplasia (HGPIN), which was reported as 4+3=7 after immunohistochemistry (IHC). The lesion accounted for 2% of the tissue strips, among which 4 score components accounted for 60% (1.8% of the tissue strips) and 3 score components accounted for 40% (0.8% of the tissue strips). |

All correct: The number of cases correctly classified by all ML models; Partial correctness: The number of cases correctly classified by some of the ML models; All misjudged: The number of cases that were misclassified by all ML models
